# Exploring the Contribution of the Transporter AGT1/rBAT in Cystinuria Progression: Insights from Mouse Models and a Retrospective Cohort Study

**DOI:** 10.3390/ijms242417140

**Published:** 2023-12-05

**Authors:** Clara Mayayo-Vallverdú, Esther Prat, Marta Vecino-Pérez, Laura González, Silvia Gràcia-Garcia, Luz San Miguel, Noelia Lopera, Angela Arias, Rafael Artuch, Miguel López de Heredia, Carlos Torrecilla, Ferran Rousaud-Barón, Oriol Angerri, Ekaitz Errasti-Murugarren, Virginia Nunes

**Affiliations:** 1 Human Molecular Genetics Laboratory, Gene, Disease and Therapy Program, Institut d’Investigació Biomèdica de Bellvitge (IDIBELL), 08908 L’Hospitalet de Llobregat, Spain; cmayayo@idibell.cat (C.M.-V.); e.prat@ub.edu (E.P.); mvecino@idibell.cat (M.V.-P.); lgsimarro@recerca.clinic.cat (L.G.); mlopezheredia@ciberer.es (M.L.d.H.); 2 Genetics Section, Physiological Sciences Department, Health Sciences and Medicine Faculty, University of Barcelona, 08907 L’Hospitalet de Llobregat, Spain; 3 Centro de Investigación Biomédica en Red de Enfermedades Raras (CIBERER), Instituto de Salud Carlos III, 28029 Madrid, Spainrafael.artuch@sjd.es (R.A.); 4 Urinary Lithiasis Laboratory, Fundació Puigvert, 08025 Barcelona, Spain; sgracia@fundacio-puigvert.es (S.G.-G.); nlopera@fundacio-puigvert.es (N.L.); 5 Urology Service, Fundació Puigvert, 08025 Barcelona, Spain; lsanmiguel@fundacio-puigvert.es (L.S.M.); frousaud@fundacio-puigvert.es (F.R.-B.); oangerri@fundacio-puigvert.es (O.A.); 6 Clinical Biochemistry Department, Institut de Recerca Sant Joan de Déu, Hospital Sant Joan de Déu, 08950 Esplugues de Llobregat, Spain; 7 Urology Service, Hospital Universitari de Bellvitge, 08908 L’Hospitalet de Llobregat, Spain; ctorrecilla@bellvitgehospital.cat

**Keywords:** cystinuria, cystine transporter, rare disease, *SLC7A13* variants, functional analysis

## Abstract

More than 20 years have passed since the identification of *SLC3A1* and *SLC7A9* as causative genes for cystinuria. However, cystinuria patients exhibit significant variability in the age of lithiasis onset, recurrence, and response to treatment, suggesting the presence of modulatory factors influencing cystinuria severity. In 2016, a second renal cystine transporter, AGT1, encoded by the *SLC7A13* gene, was discovered. Although it was discarded as a causative gene for cystinuria, its possible effect as a modulatory gene remains unexplored. Thus, we analyzed its function in mouse models of cystinuria, screened the *SLC7A13* gene in 34 patients with different lithiasic phenotypes, and functionally characterized the identified variants. Mice results showed that AGT1/rBAT may have a protective role against cystine lithiasis. In addition, among the four missense variants detected in patients, two exhibited a 25% impairment in AGT1/rBAT transport. However, no correlation between *SLC7A13* genotypes and lithiasis phenotypes was observed in patients, probably because these variants were found in heterozygous states. In conclusion, our results, consistent with a previous study, suggest that AGT1/rBAT does not have a relevant effect on cystinuria patients, although an impact in patients carrying homozygous pathogenic variants cannot be discarded.

## 1. Introduction

Cystinuria, with a worldwide prevalence of 1 in 7000 individuals, is the most common primary inherited aminoaciduria [1]. This disorder is characterized by urine hyperexcretion of cystine and dibasic amino acids, and its clinical manifestation is cystine lithiasis within the urinary system [2]. Cystine is poorly soluble at the physiological pH of urine [3], resulting in its precipitation, crystallization, and subsequent stone formation. Cystine stones account for 1–2% and 6–8% of lithiasis in adult and pediatric populations [4,5], respectively. Moreover, the high recurrence rate of stone episodes, involving frequent urologic interventions, leads to chronic kidney disease for the majority of patients [6,7].

Genetic variants in the *SLC3A1* [8] and *SLC7A9* [9] genes underlie cystinuria, as they encode the heavy (rBAT) and the light (b0,+AT) subunits of the cystine and dibasic amino acid transport system b^0,+^ [10]. The inheritance patterns differ between these genes: *SLC3A1* mutations follow an autosomal recessive pattern, whereas the *SLC7A9* gene exhibits an autosomal dominant with an incomplete penetrance pattern [6]. However, a clear genotype–phenotype correlation concerning stone onset, recurrence, and response to treatment is lacking, even among siblings with identical mutations [11,12,13]. Furthermore, while mutation analyses can identify genetic variants in the majority of patients, a notable 5–10% remain genetically uncharacterized [11,14,15].

Thus, the lack of genotype–phenotype correlation in patients with cystinuria and the finding of uncharacterized patients justifies the search for cystinuria-modulating genes.

The transport system b^0,+^ is expressed in the first segment of the proximal tubules, where it is responsible for 90% of cystine reabsorption [16]. Nevertheless, the increased expression of the heavy subunit rBAT found in the third segment of proximal tubules implies the existence of an additional light subunit capable of heterodimerizing with rBAT [17,18]. In 2016, Nagamori et al. identified AGT1, encoded by the *SLC7A13* gene, as the missing partner of rBAT. Interestingly, in vitro studies revealed that AGT1/rBAT reabsorbs cystine in exchange for aspartate and glutamate [19]. Therefore, a second cystine transporter was identified, and it was claimed as a potential cystinuria-modulating factor.

In 2018, Olschok et al. screened the *SLC7A13* gene in a cohort of 17 cystinuria patients without detected mutations in the *SLC3A1* and *SLC7A9* genes. As no pathogenic variants were found in the *SLC7A13* gene, they concluded that the novel cystine transporter AGT1/rBAT was not a third cystinuria gene [15]. However, although it may not be a causal gene for cystinuria, it could still exert a modulatory influence on the onset, severity, and progression of the disease. Thus, to explore the potential contribution of the AGT1/rBAT transporter in the progression of cystinuria, we assessed its function in cystinuria mouse models, sequenced patients exhibiting diverse lithiasic phenotypes, and functionally characterized the identified variants.

## 2. Results

### 2.1. AGT1/rBAT Contribution to Cystinuria Progression in Mouse Models

**Figure 1:**
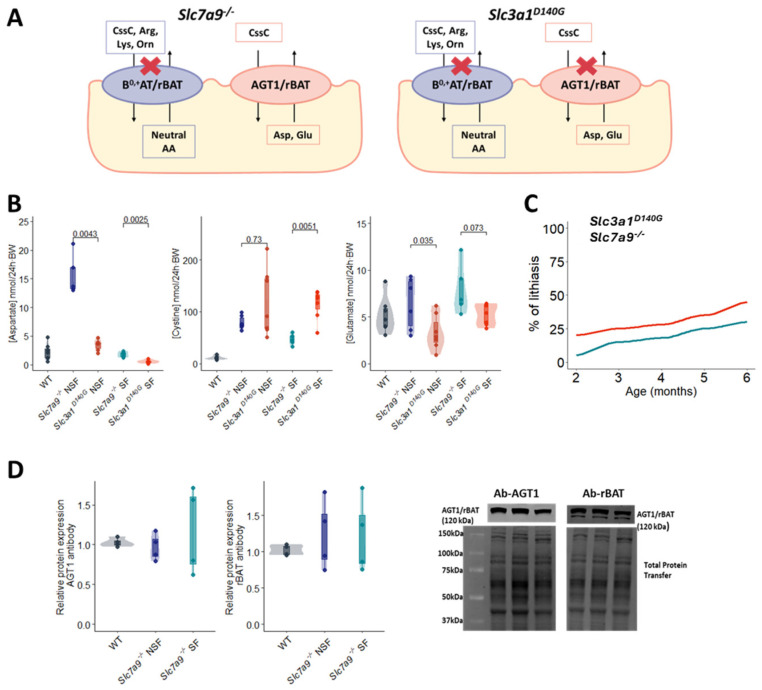
AGT1/rBAT analysis on cystinuria mouse models. (**A**) Schematic representation of the expression of renal cystine transporters in the cystinuria male mouse models. CssC = Cystine, Arg = Arginine, Lys = Lysine, Orn = Ornithine, Asp = Aspartate, Glu = Glutamate, and AA = amino acids. (**B**) Violin plot of aspartate, cystine, and glutamate urinary excretion in wild-type (WT), *Slc7a9^−/−^*, and *Slc3a1^D140G^* male mice grouped according to their lithiasic phenotype: non-stone former (NSF) and stone former (SF) mice. Urinary excretion was normalized by body weight (BW). (**C**) Monthly follow-up of the rate of stone formation in *Slc7a9^−/−^* and *Slc3a1^D140G^* male mice (N = 30 per condition). (**D**) Violin plot of AGT1/rBAT transporter expression in wild-type (WT), *Slc7a9^−/−^* non-stone former, and *Slc7a9^−/−^* stone former mice assessed using both AGT1 and rBAT antibodies in non-denaturing conditions. Representative membranes of AGT1/rBAT transporter protein levels and the total protein transference used to normalize data obtained. *p*-Values were assessed using the Mann–Whitney–Wilcoxon test.

As both cystine transporters b^0,+^AT/rBAT and AGT1/rBAT share the same heavy subunit, the *Slc3a1^D140G^* mouse model has impaired both transporters, while in the *Slc7a9^−/−^* mouse model, the AGT1/rBAT expression is preserved (Figure 1A). Thus, to assess the function of the cystine transporter AGT1/rBAT, the urinary excretion of cystine, aspartate, and glutamate was compared between *Slc7a9^−/−^* and *Slc3a1^D140G^* male mice. *Slc3a1^D140G^* mice showed higher levels of cystine and lower levels of aspartate and glutamate excretion compared to *Slc7a9^−/−^* mice in both stone and non-stone former groups (Figure 1B), evidencing the transport function of AGT1/rBAT. In addition, the stone onset in each colony was evaluated monthly for six months, and the percentage of stone former mice was lower in the *Slc7a9^−/−^* colony in the whole follow-up (Figure 1C), suggesting a protective role of AGT1/rBAT against cystine lithiasis, delaying its onset. Finally, to explore the involvement of AGT1/rBAT in cystine lithiasis, the expression of the heterodimer was compared between the non-stone former and stone former male mice of the *Slc7a9^−/−^* colony. However, no differences were observed when using either an antibody against AGT1 or rBAT (Figure 1D).

### 2.2. SLC3A1, SLC7A9, and SLC7A13 Genetic Analysis in Cystinuria Patients

First, the *SLC3A1* and *SLC7A9* genes were screened for cystinuria-causing mutations in the 34 patients recruited for this study. As shown in Table 1, we detected two mutations in the *SLC3A1* gene in fifteen patients, two mutations in the *SLC7A9* gene in eleven patients, one mutation in the *SLC7A9* gene in six patients, and two mutations in each gene in one patient. Thus, we could identify the cystinuria-causing mutations in 97% of our cohort. However, in one patient, we could not detect any causal mutation, but her high urinary levels of the four amino acids related to cystinuria indicated that we were missing something (Supplementary Table S1). Then, the *SLC7A13* gene was sequenced in all patients, and four missense variants were found (Table 2 and Supplementary Figure S1). Two of them, c.1139G>A (p.R380K) and c.1355T>C (p.M452T), had a high allele frequency in the non-Finnish European population and were predicted to be benign by the in-silico meta-predictor REVEL [20]. However, the other two variants, c.745G>A (p.V249M) and c.988C>T (p.L330F), although frequently found in the population, had an uncertain impact [21]. In addition, *SLC7A13* variants were identified as heterozygous in all patients except for one individual in whom we observed the c.745G>A variant in a homozygous state.

### 2.3. Functional Studies of SLC7A13 Missense Variants Identified in Cystinuria Patients

**Figure 2:**
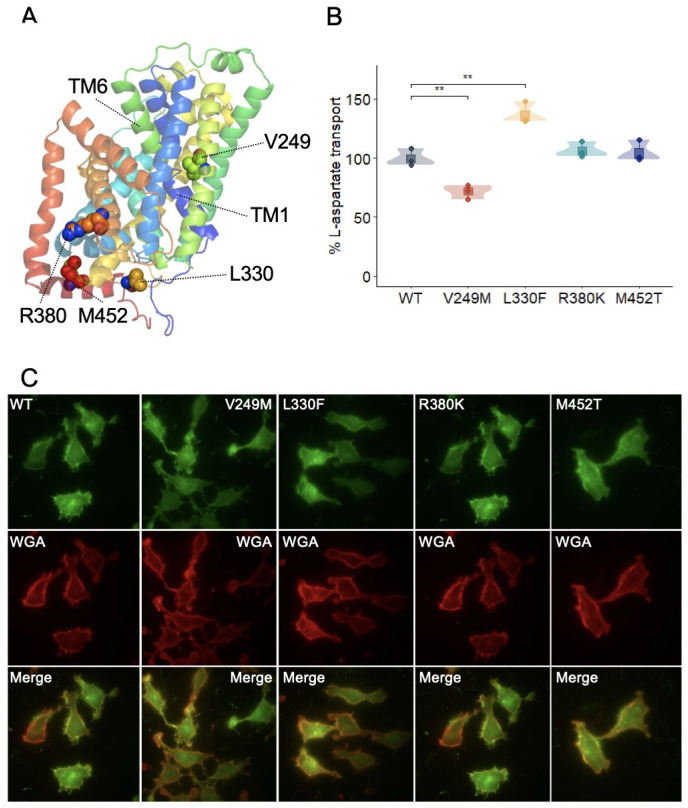
In vitro characterization of AGT1 mutants. (**A**) Cartoon representation of the AGT1 Alphafold structural model (Q8TCU3). Helices are colored blue to red from the N-terminus. Mutated residues identified in cystinuria patients are represented by spheres. Atoms are colored as follows: O (red), N (blue), and C-atoms depending on the residue (V249: green; L330 and R380: orange; M452: red). Unwound regions of TMs 1 and 6 are also depicted and indicated. (**B**) Sodium-independent L-[^3^H]-aspartate uptake mediated by human AGT1 wild-type (WT) and mutants in transfected HeLa cells. AGT1 mutant transport activity (pmols L-aspartate · mg protein · minute) is normalized to that of wild-type AGT1. Data (mean ± SEM) correspond to quadruplicates from three independent experiments. Unpaired Student’s *t*-test statistical analysis of cystinuria mutants’ activity compared to that of the wild-type counterpart is represented, *p*-value: ** ≤0.01. (**C**) Representative fluorescence microscopy images of GFP-tagged wild-type (WT) and the indicated AGT1 (green) mutants overexpressed in HeLa cells, membrane staining with wheat germ agglutinin (WGA: red), and the merged images. All AGT1 mutants reached the plasma membrane.

The Alphafold structural model of human AGT1 (Q8TCU3) was used to localize all the missense mutations identified in our cohort (Figure 2A). To functionally characterize these cystinuria variants, sodium-independent L-[^3^H]-aspartate uptake was measured in HeLa cells co-transfected with the heavy subunit rBAT and untagged wild-type AGT1 or mutants (Figure 2B). Co-expression of the heavy (rBAT) and the light (AGT1) subunits increases heterodimer plasma membrane localization [19]. All tested variants showed plasma membrane localization and expression levels comparable to those of the wild type (Figure 2C). Among the identified sequence variants, the c.745G>A (p.V249M) results are of particular interest. Valine 249 in transmembrane domain 7 (TM7) points towards the unwound region of TMs 1 and 6 (Figure 2A), which represent the core of the substrate binding site in L-amino Acid Transporters (LATs) [22], suggesting that valine to methionine substitution may affect AGT1 substrate binding site, and thus, transporter activity. In agreement, sodium-independent L-[^3^H]-aspartate uptake was significantly reduced in the V249M mutant compared to the wild-type counterpart (Figure 2B). In contrast, the L330F mutant showed an increased amino acid uptake activity, although the molecular bases associated with this gain-of-function phenotype are at present unknown. On the other hand, R380K and M452T variants did not show significant differences in L-[^3^H]-aspartate uptake when compared to the wild-type AGT1 (Figure 2B), although an effect of these mutations on cystine transport activity cannot be ruled out.

### 2.4. c.745G>A and c.988C>T SLC7A13 Variants Contribution to Amino Acid Excretion in Cystinuria Patients

**Figure 3:**
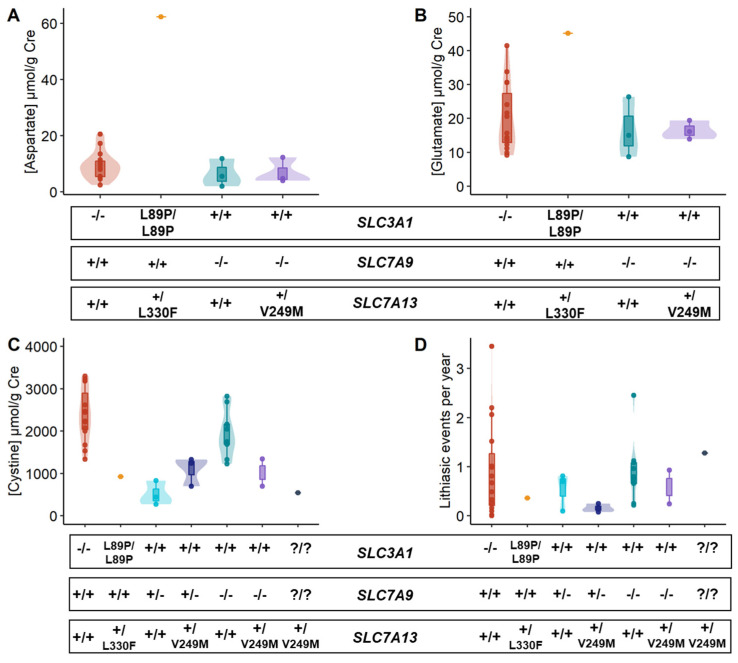
Study of the impact of c.745G>A and c.988C>T *SLC7A13* variants in cystinuria patients. Violin plots of (**A**) aspartate, (**B**) glutamate, and (**C**) cystine urinary excretion in cystinuria patients according to their cystinuria-affected gene and the carrying *SLC7A13* variant. In aspartate and glutamate comparisons, as their transport is independent of the *SLC7A9* gene and the number of samples was lower, heterozygous and compound heterozygous *SLC7A9* patients were grouped together. (**D**) Comparison of the number of lithiasic events per year of each patient according to their cystinuria-affected gene and the carrying *SLC7A13* variant. +/+ = no pathogenic variants, −/− = one pathogenic variant in each allele, +/− = one pathogenic variant in one allele, and ?/? = no pathogenic variants identified. Cre = creatinine.

After characterizing the transport function of the four missense *SLC7A13* variants, the effect of c.745G>A (p.V249M) and c.988C>T (p.L330F) variants on aspartate, glutamate, and cystine excretion in cystinuria patients was analyzed (Figure 3A–C). Unfortunately, aspartate and glutamate excretion could not be obtained from all patients. In addition, as patients with cystinuria mutations in the *SLC3A1* gene have impaired the assembly of both cystine transporters, these patients were grouped without considering *SLC7A13* variants. Only the patient with the homozygous mutation c.266C>T (p.L89P) in *SLC3A1* was analyzed separately, as previous studies on the b^0,+^AT/rBAT transporter showed a small amount of L89P mutated heterodimers reaching the membrane [23]. In the patient carrying the *SLC7A13* c.988C>T (p.L330F) variant, we observed the highest levels of aspartate and glutamate excretion of the cohort together with low levels of cystine excretion (Figure 3A–C), confirming the gain of function of this variant. However, in patients carrying the variant c.745G>A (p.V249M), no significant differences were observed regarding aspartate, glutamate, and cystine excretion (Figure 3A–C). Moreover, no influence on the number of lithiasis events per year was observed (Figure 3D), suggesting that carrying the c.745G>A (p.V249M) variant in a heterozygous state does not affect cystinuria progression.

## 3. Discussion

Under physiological conditions, over 90% of cystine reabsorption occurs via the b^0,+^AT/rBAT transporter in the first segment of proximal tubules, leaving approximately the remaining 10% to be handled by AGT1/rBAT in the third segment [16]. However, there is a lack of evidence regarding the cystine reabsorbing capacity of AGT1/rBAT when urinary cystine levels dramatically increase in cystinuria. To date, no in vivo studies using cystinuria mouse models have been conducted to explore the role of AGT1 in cystinuria further. Moreover, despite being the second renal cystine transporter identified, the impact of *SLC7A13* variants in cystinuria has only been addressed in 17 genetically uncharacterized patients [15], and its potential modulatory effect has yet to be assessed. Hence, this is the first study evaluating the contribution of AGT1 in cystinuria mouse models and exploring *SLC7A13* as a cystinuria-modulating gene.

During a six-month follow-up, *Slc3a1^D140G^* (impaired b^0,+^AT/rBAT and AGT1/rBAT transporters) male mice showed a higher stone formation rate than *Slc7a9^−/−^* (active AGT1/rBAT transporter) male mice, suggesting a protective role of AGT1/rBAT in cystine lithiasis. When comparing AGT1-related amino acid excretion between the *Slc7a9^−/−^* and *Slc3a1^D140G^* male mice, lower levels of cystine excretion and higher levels of aspartate and glutamate excretion were observed in *Slc7a9^−/−^* male mice, indicating the function of AGT1/rBAT in this model. In this line, differences in aspartate levels were more pronounced than those of glutamate. This discrepancy could be attributed to the EAAC1 (*Slc1a1*) transporter, which reabsorbs aspartate and glutamate from urine in the third segment of proximal tubules [24]. Disruption of this transporter is associated with dicarboxylic aminoaciduria, characterized by hyperexcretion of aspartate and glutamate [25]. Interestingly, the hyperexcretion of glutamate is more pronounced in both mouse models and patients, indicating a higher affinity for this amino acid [25,26]. Given that EAAC1 colocalizes with AGT1/rBAT [19], preferential reabsorption of AGT1/rBAT-released glutamate over aspartate would explain our experimental urinary amino acid levels, supporting the role of AGT1/rBAT transporter in cystine uptake in *Slc7a9^−/−^* male mice. However, no differences in cystine levels were observed between non-stone former and stone former *Slc7a9^−/−^* mice. This could be attributed to the potential underestimation of cystine detection in stone former mice, as it precipitates and aggregates into crystals/stones.

Regarding AGT1/rBAT expression, no differences were observed between stone former and non-stone former *Slc7a9^−/−^* mice. Indeed, their expression was identical to that observed in wild-type mice. Interestingly, the levels of aspartate excretion were higher in *Slc7a9^−/−^* non-stone former mice compared to *Slc7a9^−/−^* stone former mice. Additionally, the levels of aspartate in wild-type mice, in which cystine reabsorption mediated by AGT1 accounts for about 10%, resemble those in *Slc3a1^D140G^* and *Slc7a9^−/−^* stone former mice. This finding suggests that the seven-fold increase in aspartate excretion in *Slc7a9^−/−^* non-stone former mice indicates that AGT1/rBAT is able to reabsorb, at least partially, excess urinary cystine by exchanging it for aspartate. In *Slc7a9^−/−^* stone former mice, glutamate urinary levels are slightly higher than those observed in wild-type, *Slc3a1^D140G^*, and *Slc7a9^−/−^* non-stone former mice, suggesting a likely AGT1/rBAT-dependent cystine reabsorption mechanism based on glutamate (instead of aspartate) exchange. However, further analyses are necessary to fully determine if AGT1/rBAT function is a crucial factor for cystine lithiasis progression in *Slc7a9^−/−^* male mice.

In 2018, Olschock et al. analyzed 17 patients without cystinuria-causing mutations in *SLC3A1* or *SLC7A9* genes. Subsequent screening of the *SLC7A13* gene in these patients revealed only benign polymorphisms, leading to the exclusion of *SLC7A13* as the third causative gene for cystinuria in their cohort [15]. However, no additional clinical parameters such as amino acid excretion levels, stone onset, or disease progression were provided to assess the impact of these polymorphisms on cystinuria pathology. In our study, *SLC3A1*, *SLC7A9,* and *SLC7A13* genes were sequenced in 34 clinically diagnosed cystinuria patients. In our cohort, only one patient remained genetically uncharacterized for the cystinuria-causing mutation in the *SLC3A1* and *SLC7A9* genes. Furthermore, four *SLC7A13* missense variants were identified. While two of them were predicted as benign (c.1139G>A (p.R380K) and c.1355T>C (p.M452T)), the other two were classified as uncertain by the in-silico meta-predictor REVEL (c.745G>A (p.V249M) and c.988C>T (p.L330F)) [20]. All variants found had allele frequencies exceeding 1%, indicating that no rare variants were identified in our cystinuria patient cohort.

To further elucidate the functional impact of these *SLC7A13* variants on the AGT1/rBAT transporter, in vitro functional analyses were conducted in HeLa cells co-transfected with the heavy subunit rBAT and untagged-AGT1, including wild-type and mutants. Results demonstrated that while R380K and M452T mutants did not affect transporter activity, the V249M and L330F mutants exhibited a 25% decrease and a 37% increase in aspartate uptake activity, respectively. The position of valine 249 in TM7, pointing towards the core of the AGT1/rBAT substrate binding site, suggests that valine-to-methionine substitution may affect AGT1 substrate binding and/or translocation, thus influencing transporter activity. In contrast, the localization of leucine 330 in the Alphafold structural model of human AGT1 provides no clues about the molecular mechanism underlying the gain of function observed in the L330F mutant.

The c.745G>A (p.V249M) variant was the most frequent in our cohort, detected in 41% of the studied patients, but only one was found in the homozygous state. Its impact was studied in patients with *SLC7A9* mutations, as AGT1/rBAT is assembled, but no significant changes in amino acid excretion or disease progression were observed. Although the number of patients should be increased, our results suggest that the c.745G>A variant in heterozygous has no obvious effect in cystinuria patients. Regarding the c.988C>T (p.L330F) variant, it was identified in only one patient and was heterozygous. Whereas in this patient, we detected a homozygous mutation in the *SLC3A1* gene (c.266C>T, p.L89P), it has been reported that a significant fraction of rBAT L89P mutant heterodimers reach the membrane and remain functional for amino acid transport [23]. For this reason, amino acid levels from this patient were analyzed separately from the rest of the *SLC3A1* patients. Interestingly, this patient showed the highest urinary levels of aspartate and glutamate of our cohort, together with low levels of cystine, reinforcing the gain of AGT1/rBAT function characterized in vitro for the L330F mutant. However, this gain of function in cystine reabsorption did not correlate with lower lithiasis events per year, suggesting that it was insufficient to avoid or delay cystine lithiasis recurrence.

In conclusion, despite results indicating an AGT1/rBAT impact on cystine lithiasis in mouse models, no cystinuria causative or modulating effect could be associated with AGT1/rBAT after screening the *SLC7A13* gene in 34 cystinuria patients. However, we could demonstrate in vitro that two *SLC7A13* variants found in patients affect amino acid excretion. Nevertheless, the lack of conclusive evidence of AGT1/rBAT significance in cystinuria patients in our cohort, coupled with similar findings observed by Olschok et al., decreases the possibility of the expected relevance of AGT1/rBAT in cystinuria. Additional research in different cohorts of cystinuria patients is essential to find rare, pathogenic, homozygous variants in *SLC7A13* to fully characterize its impact on cystinuria.

## 4. Materials and Methods

### 4.1. Mouse Proceedings

The mouse colonies were carefully maintained in the animal facility of IDIBELL under pathogen-free conditions. Animal Experimentation Ethics Committee of IDIBELL (AAALAC accredited facility, B9900010) approved all mouse procedures performed. The mice were housed in cages positioned within ventilated racks in a room with controlled temperature, humidity, and a 12 h light cycle. The provided chow was VRF1 P from Special Diets Services, UK, with the code 801900.

Homozygous *Slc7a9^−/−^* [18] and *Slc3a1^D140G^* [27] cystinuria mouse models on a C57BL/6J genetic background were used in this work. Only male mice were used in this study, as female mice have no expression of AGT1 in the kidney [19].

The follow-up of the stone onset was performed by taking monthly X-ray images using an IVIS Lumina XR Series III (Caliper LifeScience–Vertex Technics, Hopkinton, MA, USA). The acquired images were examined employing Living Image^®^ Software (v4.1). At the end of the follow-up, mice were housed in metabolic cages over a four-day period to collect their individual urine. Then, mice were euthanized, and both kidneys and stones were extracted.

Cystine, aspartate, and glutamate were determined in urine samples using UPLC–MS/MS in Dr. Rafael Artuch’s laboratory at Hospital Sant Joan de Déu, as described in [28]. Data were normalized by 24 h urinary excretion and mice body weight in grams (24 h·BW).

### 4.2. Brush-Border Western Blot

To determine transporter expression, apical brush-border membranes (BBMs) from mouse kidneys were extracted as published in [29]. Protein concentration was determined with the BCA Protein Assay Kit (ThermoScientific, Waltham, MA, USA). BBMs were separated on Stain-Free Precast Protein Gels (Bio-Rad) under non-denaturing conditions to preserve the heterodimer. Stain-free was activated for 45 s, and the semi-dry transfer was performed using the Trans-Blot^®^ Turbo™ transfer system (Bio-Rad, Hercules, CA, USA) onto a nitrocellulose membrane at 2.5 mA and 25V for 7 min. AGT1 [19] and rBAT [30] primary antibodies were incubated overnight at 4 °C. The secondary antibody (Li-cor #926-68023) was incubated for 1 h at RT. The blots were developed with the Odyssey^®^ Classic (Li–Cor), and the total protein amount loaded in the gel was determined using a ChemiDoc™ Touch (Bio-Rad, Hercules, CA, USA) with a Stain-free stain.

### 4.3. Patients

Patients enrolled in the present study were clinically diagnosed with cystinuria at Fundació Puigvert (Barcelona, Spain) or Hospital Universitari de Bellvitge (L’Hospitalet de Llobregat, Spain). Informed consent was obtained from all participants (*n* = 34) or their legal guardians to perform genetic analysis. Inclusion criteria comprised the clinical diagnosis of cystinuria, at least 5 years of disease follow-up in the same center, and the availability of urinary levels of cystine. There were no exclusion criteria for sex, age, lifestyle, or other medical conditions.

Blood samples were obtained to extract DNA to analyze genetic variants in the *SLC3A1*, *SLC7A9*, and *SLC7A13* genes. Clinical data related to cystine lithiasis were recorded from the clinical history of each patient. Thus, the number of new stone occurrences, spontaneous stone expulsions, renal colic, hematuria due to lithiasis, acute pyelonephritis, hospital admissions due to lithiasis, and surgical intervention to remove the lithiasis were grouped into a single numerical variable called lithiasis events. The number of lithiasis events for each patient was normalized by years of follow-up, as other authors have proceeded [31,32]. Urinary excretion of cystine, lysine, ornithine, and arginine, and where possible, aspartate and glutamate were also acquired. Supplementary Table S1 is a summary of the data collected and analyzed from patients.

### 4.4. Patient Genotyping

The Wizard^®^ Genomic DNA Purification Kit (Promega, Madison, WI, USA) was used to extract DNA from peripheral blood samples. Then, PCR was conducted to amplify each exon of the *SLC3A1, SLC7A9*, and *SLC7A13* genes using the primers described in Supplementary Table S2. Sanger Sequencing Analysis was conducted by Stab Vida Company (Portugal), and the Sequencher software (v5.4) was used to assemble the sequences and identify variants.

NG_008233.1 (*SLC3A1*), NG_001126335.2 (*SLC7A9*), and NC_000008.10 (*SLC7A13*) were used as reference sequences. Large deletions and duplications were detected performing an MLPA assay with specific probemixes for cystinuria (SALSA^®^ MLPA^®^ probemix P426-A1 Cystinuria, MRC-Holland, Amsterdam, The Netherlands). The in-silico meta-predictor REVEL was used to assess the pathogenicity of the detected variants [20]. Moreover, allele frequencies for the non-Finnish European population were obtained from gnomAD (v4.0.0).

### 4.5. DNA Constructions and Site-Directed Mutagenesis

Codon-optimized sequences of human AGT1, AGT1-GFP, and rBAT were purchased from Proteogenix (Schiltigheim, France). Point mutations in the *SLC7A13* sequence were introduced via site-directed mutagenesis using the QuikChange kit (Stratagene, La Jolla, CA, USA), following the manufacturer’s instructions. The GFP-tagged and pcDNA3.1–*SLC7A13* constructs were used as templates. Compatible reverse and forward primers were used to introduce nucleotide substitutions into the *SLC7A13* sequence. The resulting constructs were verified using DNA sequencing before transient transfection.

### 4.6. Cell Culture and Transfection

HeLa cells were maintained in Dulbecco’s Modified Eagle’s Medium (Life Technologies, Carlsbad, CA, USA) at 37 °C/5% CO_2_, supplemented with 50 units/mL penicillin, 50 μg/mL streptomycin, 10% (*v*/*v*) fetal bovine serum, and 2 mM L-glutamine. Transient transfection of HeLa cells was performed using Lipofectamine 2000 (Invitrogen, Carlsbad, CA, USA) following the manufacturer’s instructions. Amino acid transport experiments and subcellular localization by means of fluorescence microscopy were carried out 48 h after transfection.

### 4.7. Visualization of GFP-Tagged Amino Acid Transporters Using Fluorescence Microscopy

The expression and plasma membrane localization of AGT1 wild-type and mutant were analyzed using fluorescence microscopy in a semiconfluent monolayer of HeLa cells co-transfected with rBAT and GFP-tagged hAGT1. HeLa cells cultured on glass coverslips were fixed for 15 min in 4% paraformaldehyde, rinsed three times in Hank’s Balanced Salt Solution (HBSS), incubated with 1 mg/mL wheat germ agglutinin (WGA) labeled with Texas-Red (Thermo Fisher Scientific) at room temperature for 10 min, and rinsed three times with phosphate-buffered saline-Ca^2+^–Mg^2+^. Glass coverslip containing fixed and membrane-stained cells were mounted with aqua-poly/mount coverslipping medium (Polysciences Inc., Niles, IL, USA). Images were captured using a Nikon E1000 (Tokyo, Japan) upright epifluorescence microscope. All images were captured during a 200 ms exposure.

### 4.8. Amino Acid Transport Assay

Sodium-independent L-[^3^H]-labeled aspartate (1 μCi/mL; Perkin Elmer, Shelton, CT, USA) uptake was measured at room temperature in sodium-free transport buffer (137 mM choline chloride, 5 mM KCl, 2 mM CaCl_2_, 1 mM MgSO_4_, and 10 mM HEPES, pH 7.4). Initial transport rates were determined using an incubation period of 1 min and 100 µM of cold aspartate [19]. Aspartate uptake was terminated by washing with 3 mL of cold transport buffer. Cells were incubated for 1 h at 37 °C in lysis buffer (100 mM NaOH and 0.1% SDS), and radioactivity was measured using a scintillation counter.

### 4.9. Statistical Analysis

Statistical analyses and figure preparation were conducted using RStudio, version 3.6.0. The packages employed included ggplot2, openxlsx, dplyr, stringr, and devtools. Violin plots were chosen to represent data, facilitating a clearer visualization of the distribution of samples between groups. The D’Agostino & Pearson omnibus normality test was applied to assess the normality of data. Subsequently, either Mann–Whitney or t-test was used as appropriate, with details provided in the legend of each figure. A *p*-Value less than 0.05 was set as a threshold for significance, although values below 0.1 were also considered important for further discussion.

## Figures and Tables

**Table 1:** Classification of the 34 patients analyzed according to the number of mutations found in the *SLC3A1* and *SLC7A9* genes.

	Two Mutations	One Mutation	No Mutations
*SLC3A1*	15 (44%)	/	/
*SLC7A9*	11 (32%)	6 (18%)	/
Both Genes	1 (3%)	/	/
Total	27 (79%)	6 (18%)	1 (3%)

**Table 2:** *SLC7A13* variants found in 34 cystinuria patients.

*SLC7A13* Variant (NM_138817.3)	AGT1 Change (NP_620172.2)	REVEL Prediction	Allele Frequency in the Non-Finnish European Population	Nº of Alleles Found in Our Cohort	Allele Frequency in Our Cohort	Zygosity in Our Cohort
c.745G>A	p.V249M	0.291 (Uncertain)	0.151	15	0.221	13 heterozygous1 homozygous
c.988C>T	p.L330F	0.355 (Uncertain)	0.030	1	0.015	1 heterozygous
c.1139G>A	p.R380K	0.162 (Benign)	0.128	11	0.162	11 heterozygous
c.1355T>C	p.M452T	0.156 (Benign)	0.086	4	0.059	4 heterozygous

## Data Availability

Data that are presented in this study are available upon request from the corresponding authors.

## Supplementary Materials

The supporting information can be downloaded at https://www.mdpi.com/article/10.3390/ijms242417140/s1.
