# Supplementary material for: Exploring the Contribution of the Transporter AGT1/rBAT in Cystinuria Progression: Insights from Mouse Models and a Retrospective Cohort Study
Source: Int J Mol Sci. 2023 Dec 5;24(24):17140. doi: 10.3390/ijms242417140 (PMC10743100; doi:10.3390/ijms242417140)
Supplement: Supplementary file 1 [file ijms-24-17140-s001.zip › ijms-2721298-supplementary.pdf]

Supplementary Figure S1. 2D structural representation of human AGT1. Secondary structure prediction of hAGT1 sequence. Amino acid positions mutated in cystinuria patients are indicated.

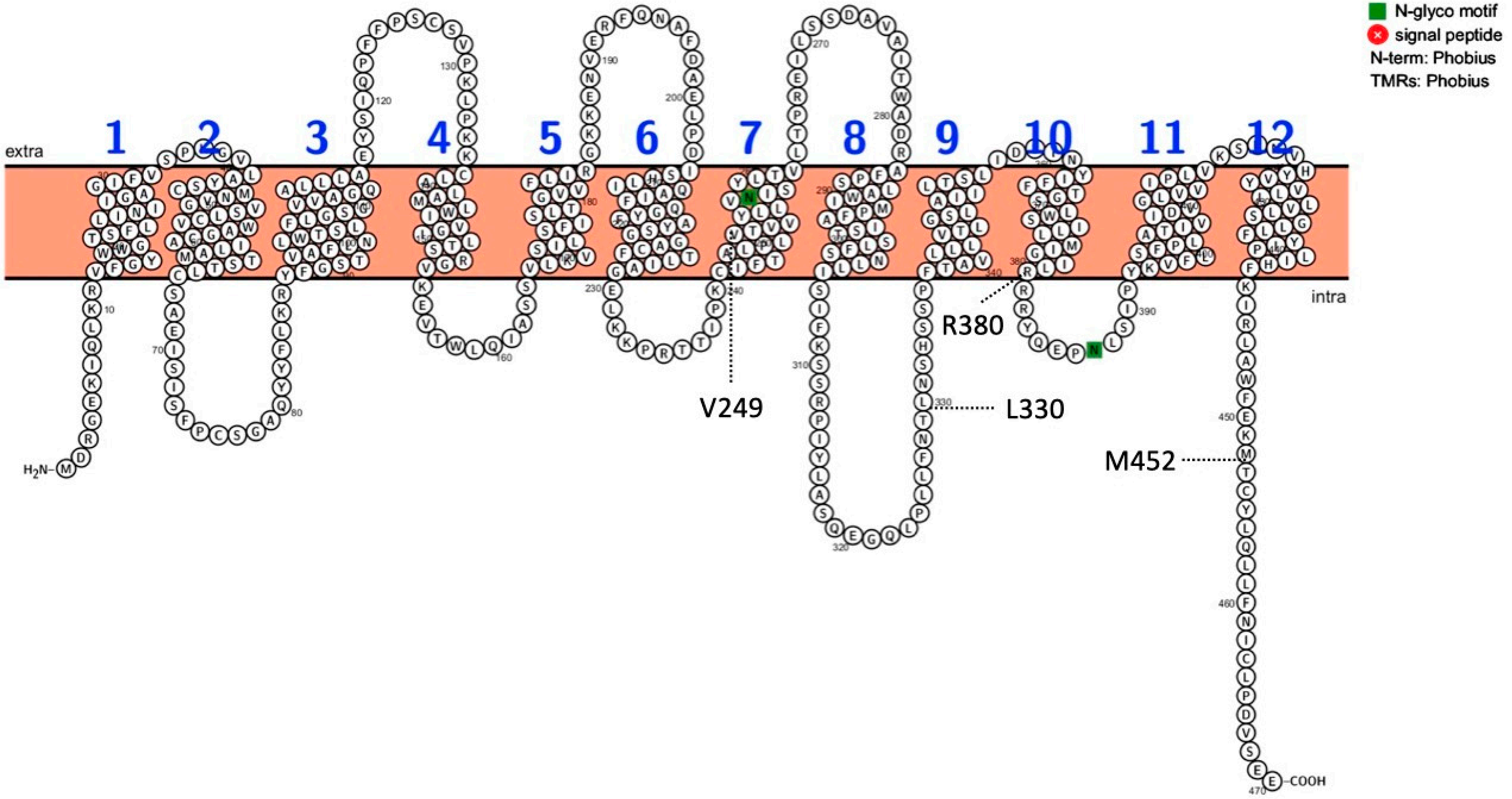

supplementary Table S1. Sumary of patients' data coll. CssC = Cystine, Asp = Aspartate, Glu = Glutamate, Lys = Lysine, Arg = Arginine, Orn = Ornithine.

ID	Cystinuria Gene	Variants	Protein change	*SLC7A13* Variants	AGT1 change	Sex	Age	Age at diagnostic	Lithiasic Episodes	CssC	Asp	Glu	Lys	Arg	Orn
**1**	*SLC3A1*	c.1093C>T/c.1400T>C	p.R365W/p.M467T	-		male	38	14	3.45	2446	17.2	24.1	5274	1769	1736
**2**	*SLC3A1*	c.1093C>T/c.1400T>C	p.R365W/p.M467T	-		male	35	11	1.52	2441	20.6	30.6	8879	2371	1673
**3**	*SLC3A1*	c.1400T>C/c.1400T>C	p.M467T/p.M467T	c.745G>A	p.V249M	male	70	25	0.67	1338	8.3	15.6	2867	701	780
**4**	*SLC3A1*	c.1400T>C/c.1400T>C	p.M467T/p.M467T	c.745G>A	p.V249M	female	64	18	0.02	2003	8,00	30.7	4559	569	918
**5**	*SLC3A1*	c.1354C>T/DupE5-E9	p.R452W/DupE5-E9	c.1139G>A	p.R380L	male	63	53	0.99	1671	9.5	33.8	3385	1141	1192
**6**	*SLC3A1*	c.266T>C /c.266T>C	p.L89P/p.L89P	c.745G>A /c.988C>T	p.V249M/ p.L330F	female	46	34	0.36	925	62.4	45.1	7499	3684	1950
**7**	*SLC3A1*	DelE1-E6 /DelE1-E6	DelE1-E6/DelE1-E6	c.1139G>A	p.R380L	female	67	25	0.1	3180	13.5	41.5	5849	3735	1211
**8**	*SLC3A1*	c.1400T>C/DupE5-E9	p.M467T/DupE5-E9	c.745G>A	p.V249M	female	55	21	0.16	3297	5.1	12.3	5849	2130	1152
**9**	*SLC3A1*	c.1354C>T/DupE5-E9	p.R452W/DupE5-E9	c.1139G>A	p.R380L	female	53	41	0.26	3186	9.7	21.6	7351	3661	1367
**10**	*SLC3A1*	c.431G>C/c.431G>C	p.G144A/p.G144A	c.1139G>A	p.R380L	male	41	33	2.2	2216	5.5	9.9	5545	4433	1277
**11**	*SLC3A1*	c.1400T>C/DupE5-E9	p.M467T/DupE5-E9	c.745G>A	p.V249M	male	22	17	2.06	2078	4.5	9.1	6336	1876	1144
**12**	*SLC3A1*	c.647C>T/c.647C>T	p.T216M/p.T216M	-		female	62	12	0.49	2617	11.4	14.4	4414	4884	876
**13**	*SLC3A1*	c.1400T>C/DupE5-E9	p.M467T/DupE5-E9	c.745G>A/ c.1355T>C	p.V249M/ p.M452T	male	79	30	0.33	1532	8.5	13.5	4210	2605	1002
**14**	*SLC3A1*	c.1400T>C/c.1400T>C	p.M467T/p.M467T	c.1139G>A	p.R380L	male	48	13	0	2470	2.4	11.2	5938	2098	1234
**15**	*SLC3A1*	c.754C>T/c.754C>T	p.P252S/p.P252S	c.745G>A	p.V249M	male	52	19	0.81	2233	5.3	14.2	5125	2903	1803
**16**	*SLC3A1 & SLC7A9*	c.1139delT/c.1354C>T c.368C>T/c.578_580delTCA	p.L380fs/p.R452W p.I193del/p.T123M	c.745G>A	p.V249M	male	40	15	1.01	3229	10.6	20.6	4806	499	985
**17**	*SLC7A9*	c.368C>T/-	p.T123M/-	c.745G>A/ c.1139G>A	p.V249M/ p.R380L	female	35	27	0.25	1243	4.8	13.9	1860	75	206
**18**	*SLC7A9*	c.368C>T/c.997C>T	p.R333W/p.T123M	c.1355T>C	p.M452T	female	39	21	0.74	1689	2,00	8.7	1987	79	333
**19**	*SLC7A9*	c.1367G>A/-	p.R456H/-	c.1139G>A	p.R380L	female	70	31	0.7	271	NA	NA	2040	130	250
**20**	*SLC7A9*	c.311G>A/c.311G>A	p.G105R/p.G105R			female	73	30	0.22	1223	NA	NA	5670	2178	969
**21**	*SLC7A9*	c.768G>A/-	p.G195R/-			female	58	NA	0.1	829	NA	NA	2241	91	231
**22**	*SLC7A9*	c.614dupA/-	p.N206Efs*3/-	c.1355T>C	p.M452T	male	46	NA	0.81	441	NA	NA	2450	123	274
**23**	*SLC7A9*	c.614dupA/c.544G>A	p.N206Efs*3/p.A182T			female	43	NA	1.12	1753	NA	NA	5678	269	380
**24**	*SLC7A9*	c.579_581dupCAT/-	p.I193dup/-	c.745G>A	p.V249M	male	37	NA	0.15	1331	12.3	16.1	5870	265	489
**25**	*SLC7A9*	c.614dupA/-	p.N206Efs*3/-	c.745G>A	p.V249M	male	32	10	0.08	697	NA	NA	3356	166	200
**26**	*SLC7A9*	c.313G>A/c.368C>T	p.G105R/p.T123M	c.1139G>A	p.R380L	male	30	3	0.96	2692	NA	NA	2280	111	304
**27**	*SLC7A9*	c.368C>T/c.614dupA	p.T123M/ p.N206Efd*3	c.745G>A	p.V249M	male	44	19	0.24	1345	4.0	19.4	1005	96	120
**28**	*SLC7A9*	c.614dupA/c.844G>A	p.N206Efs*3/p.E282K	c.1139G>A	p.R380L	male	24	2	0.66	2047	NA	NA	1790	3096	5153
**29**	*SLC7A9*	c.614dupA/c.997C>T	p.N206Efs*3/ p.R333W			male	65	NA	0.25	2821	11.87	26.35	7314	556	227
**30**	*SLC7A9*	c.997C>T/skippingE3	p.R333W/skipping E3	c.1139G>A	p.R380L	female	67	37	1.08	2166	5.5	15.0	7336	2846	1405
**31**	*SLC7A9*	c.544G>A/DupE12	p.A182T/DupE12	c.745G>A	p.V249M	female	42	18	0.93	700	NA	NA	859	370	226
**32**	*SLC7A9*	c.1397C>A/c.368C>T	p.S466*/p.T123M	c.1139G>A	p.R380L	male	44	28	2.45	1326	NA	NA	NA	NA	NA
**33**	*SLC7A9*	c.1397C>A/c.368C>T	p.S466*/p.T123M	c.1355T>C	p.M452T	male	34	18	0.8	1722	NA	NA	NA	NA	NA
**34**	*?*	-	-	c.745G>A/ c.745G>A	p.V249M/ p.V249M	female	Exitus	NA	1.28	545	NA	NA	1349	168	435

Supplementary Table S2. Primers used to genotype patient samples. *Large exons were divided in two parts, A and B. F = Forward and R = Reverse.*

SLC3A1	**Exon**	**Sequence**
	E1-F	CTTTCTTCCTTGGCTGGACT
	E1-R	CTGAACAACCCAGGCATAAT
	E2-F	TACAGGCGTGAACCACTACA
	E2-R	ATCTTGCCCACTTTCCATTC
	E3-F	GCCTGGCCTGTCATATGTTAT
	E3-R	GGGTTTTACTAAATCAGTTCAATCA
	E4-F	TGTCCATTTCTGTGAAACACC
	E4-R	TCAAATAATTAAAGACTTGATTTTGC
	E5-F	TGCCAAGTTGTTAACAGTCAAA
	E5-R	TCAGGCTGAGAAAGAAAACAC
	E6-F	GAGCCCTTTGAAGAGGTTGT
	E6-R	CCTCCTACAGTGCTGGGTTT
	E7-F	ATGCTATCCTTCCCTTAGCC
	E7-R	CATTTTAGAGATAACTGGACAGCA
	E8-F	TTGCTACGTTGTGAACTTTCTG
	E8-9	CATGATTTTCAGCAATGCAA
	E9-F	GGGTGAAACTGGTTTATGTACCG
	E9-R	ACTTCACTTCACTTGGTAGATTTGT
	E10-F	GGATCGAGTGTTTTGGGTAAAT
	E10-R	CCAAGCAGCATGCTGTACAT
SLC7A9	**Exon**	**Sequence**
	E1-F	CATTTCTAGGGTTGGACCGTG
	E1-R	GGCCAGGAGAGCCATGAG
	E2-F	ATGACTGACTTTGACTCTGGG
	E2-R	TCTTCTGCCGTGTCACTAGGG
	E3-F	CGCCCTCTTCCTTCCTCC
	E3-R	TAGCAGCTGCCTGGCGTG
	E4-F	ACCCTGCCAGTATCCCTCTT
	E4-R	CAGAGACTCACTGGGGAGGA
	E5/6-F	TCCCGTGGAGATACACTCA
	E5/6-R	TGGAGTTAAAGTCACCTGGAG
	E7-F	AGTCAAGGTGTGTGACGCTTG
	E7-R	AGGAGAAGAGAAATCAGGCTG

	E8-F	CTGAACGTGGGTCTCCGTG
	E8-9	ACCTCCAGTGCTGACACCTG
	E9-F	ACCTCCTCACTCACTCTGCC
	E9-R	TCATAGCAAGGAATAAGGGCATCT
	E10-F	GGAGCACAAGTCCTCAGTGG
	E10-R	GCCTTGAAGATAGGCTGGTAG
	E11-F	TTCGGTCTTCTGTGACATGAG
	E11-R	GTCAGATTGGAACTAGAAGGCA
	E12-F	ATGATTGAAATTGGAGGAGGG
	E12-R	TGGAGTCAGGACAGGTGAGG
	E13-F	CCTCACCCACAACAACTCC
	E13-R	AAATTCAGCTGACTTGGCTAC
SLC7A13	**Exon**	**Sequence**
	1A-R	GACAGAGCAGCTGGGAAAAAAAGG
	1A-F	CGAAAATGGAACAGGAAGGACAGAA
	1B-R	CCAGGCTGGCATGATCTGATTC
	1B-F	TTTGGCTCCACGGTTGCTTT
	2-R	TCATTCCCTTTGGCCCTCTGT
	2-F	TGACCACCACACAACCTATCCACA
	3-R	CTCATGTGTTTCACAGTAACTGAGTA
	3-F	TTTTGTTTTTCTGCAGATGCTGTAG
	4A-R	CATGCGAAGCAGAGCTTTTAGC
	4A-F	TGCAGGTATCATTCATGGATGTTC
	4B-R	AAGTGCTACATTCTGGAAGGGAAAA
	4B-F	TAATATTTGCCTCCCTGATGTGTCT
